# A Young Male with Severe Myocarditis and Skeletal Muscle Myositis

**DOI:** 10.1155/2018/5698739

**Published:** 2018-06-14

**Authors:** Abdalla Ibrahim, Eoghan Meagher, Alexander Fraser, Thomas J. Kiernan

**Affiliations:** ^1^Cardiology Department, University Hospital Limerick, St. Nessan's Road, Dooradoyle, Limerick, Ireland; ^2^Rheumatology Department, University Hospital Limerick, St Nessan's Road, Dooradoyle, Limerick, Ireland

## Abstract

A 34-year-old male presented with retrosternal chest pain, fatigue, shortness of breath, and a history of a previous episode of myocarditis four years prior. He had elevated troponin T, normal skeletal muscle enzymes, and negative inflammatory markers. Cardiac magnetic resonance imaging (MRI) confirmed active myocarditis with extensive myocardial fibrosis and normal left ventricular ejection fraction (LVEF). His myocarditis symptoms resolved with steroids and anti-inflammatory treatment, but on closer questioning, he reported a vague history of long-standing calf discomfort associated with episodes of stiffness, fatigue, and flu-like symptoms. MRI of the lower legs consequently demonstrated active myositis in the calf muscles. Immunomodulatory therapy was commenced with good effect. The patient is undergoing regular follow-up in both cardiology and rheumatology outpatient departments. Repeated MRI of the legs showed significant interval improvement in his skeletal muscle myositis, and repeat cardiac MRI demonstrated the resolution of myocarditis along with persistent stable extensive myocardial fibrosis and preserved LVEF. The patient has returned to full-time work.

## 1. Introduction

The diagnosis of myocarditis remains challenging given the diverse nature of clinical presentations [1]. The exact incidence is difficult to determine since endomyocardial biopsy, the gold standard diagnostic test, is used infrequently [2]. It has been reported that as few as 10% of people with polymyositis present with a cardiac involvement as the initial feature of the disease compared to 70% of patients with known polymyositis who experience some cardiac involvement as their disease progresses [3]. Clinically significant cardiac involvement has rarely been reported in myositis patients [4], while subclinical manifestations more commonly present as conduction abnormalities and arrhythmias detected on electrocardiography (ECG) [5].

## 2. Case Presentation

**Figure 1:**
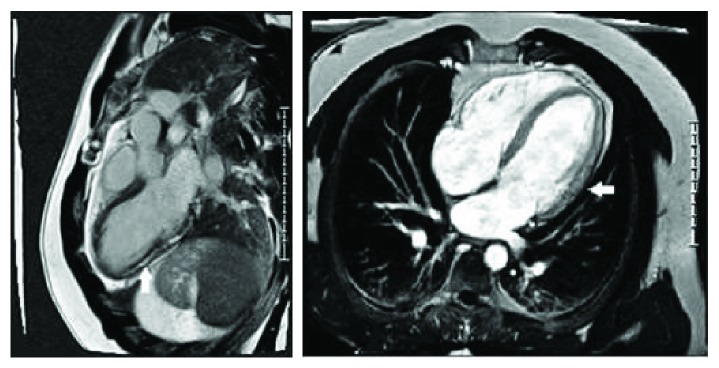
Cardiac MRI showing extensive subepicardial and midwall late enhancement typical of myocarditis in the anterior, lateral, and inferior walls.

A 34-year-old male was admitted to the hospital with recurrent episodes of retrosternal chest pain, fatigue, and shortness of breath with an elevated troponin T. He had suffered an acute episode of myocarditis four years previously requiring hospital admission. He had no other relevant medical history and no family history of cardiac disease. He is a nonsmoker and consumed alcohol occasionally. Clinical examination was unremarkable and did not show any evidence of heart failure or systemic disease. ECG showed normal sinus rhythm without any ischemic changes, and chest X-ray showed no evidence of infection or heart failure. Routine blood tests including antinuclear antibody, creatinine kinase (CK), rheumatoid factor, and C-reactive protein were all within normal limits apart from an elevated cardiac troponin T with a peak value of 2700 ng/l (<14 ng/l). Further extensive inflammatory, viral, and autoimmune screening was carried out and found to be negative. Subsequent coronary angiogram showed normal coronary arteries, and transthoracic echocardiography demonstrated left ventricular ejection fraction (LVEF) >55% with trace mitral regurgitation. Cardiac magnetic resonance imaging (MRI) demonstrated extensive subepicardial and midwall late enhancement typical of myocarditis in the anterior, lateral, and inferior walls along with extensive fibrosis with normal LVEF (Figure 1).

**Figure 2:**
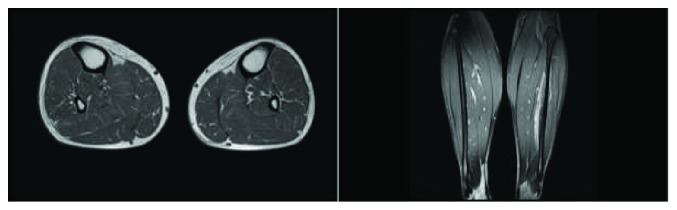
MRI of the left leg showing patchy increased STIR signal in the muscles but predominately involving the gastrocnemius muscles.

**Figure 3:**
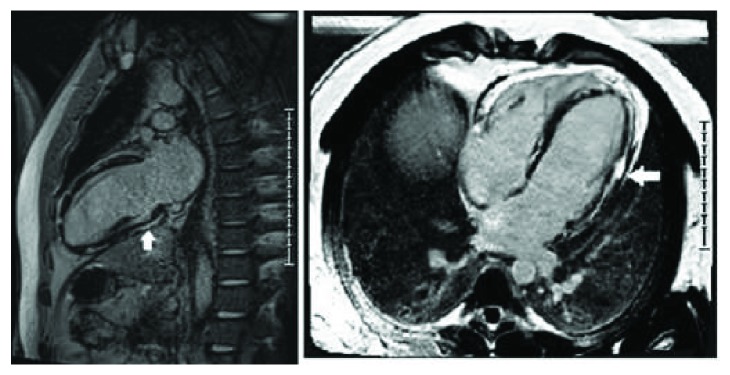
Cardiac MRI showing extensive epicardial and midwall fibrosis in the anterolateral, lateral, inferolateral, and inferior walls from base to apex.

A short course of steroids and anti-inflammatory medication as an inpatient resulted in the resolution of his myocarditis symptoms. The troponin T level normalized and the patient was discharged with a plan to repeat cardiac MRI in six months. On follow-up as an outpatient, it was decided to refer the patient to rheumatology for an opinion regarding ongoing immunomodulatory therapy. At this juncture, the patient stated that he also had symptoms of stiffness and aching in his calf muscles for quite some time but he did not consider it to be relevant. Despite persistently normal skeletal muscle enzyme levels, an MRI of the lower legs was performed and this showed active myositis involving the gastrocnemius muscles bilaterally (Figure 2). As the patient was demonstrated to have ongoing myositis despite minimal symptoms, and as he had accrued significant myocardial scarring from previous episodes of myocarditis, it was decided to commence long-term immunomodulatory therapy in the form of methotrexate and prednisolone. Clinically, the patient reported a significant improvement in his symptoms and a repeat of the lower limb MRI demonstrated a significant interval improvement in his skeletal muscle myositis. Six months later, a repeat of the cardiac MRI demonstrated resolution of myocarditis along with persistent, stable, and extensive myocardial fibrosis and preserved LVEF (Figure 3). The patient is tolerating the immunomodulatory therapy well without major side effects, and he has returned to full-time work.

## 3. Discussion

We describe an elusive case of a young male with recurrent episodes of acute myocarditis presenting with chest pain and elevated troponin T. All biochemical, immune, and autoimmune tests were within normal limits. However, he described a long-standing and persistent history of calf aching and discomfort. Consequently, lower limb MRI revealed that the patient had active myositis of his gastrocnemius muscles. This unexpected finding demonstrated that this inflammatory myositis was not isolated to cardiac muscle and by definition was still active despite initial treatment. As this patient had already accrued significant myocardial scarring, the finding of active myositis demonstrated the need for more effective long-term immunomodulatory therapy in order to preserve cardiac function. Cardiac involvement in patients with skeletal myositis was first reported in 1899 [6]. Subclinical conduction abnormalities and arrhythmias discovered by ECG are considered to be more frequent than clinically manifest heart involvement [5]. The number of reported cases of clinically overt cardiac involvement in patients with myositis has increased recently, largely due to the increased use of noninvasive sensitive tests such as troponin [5]. The overall incidence of heart involvement in myositis patients ranges from 6% to 75% depending on the type (clinical or subclinical), definition of cardiac involvement, and tests used to confirm the diagnosis [3, 7]. Cardiovascular involvement is considered to be one of the major causes of mortality in patients with myositis [8, 9]. Mortality due to myocardial infarction in patients with myositis was reported to be increased by 16 times, especially among female patients [10]. Despite the lack of large epidemiological studies, cardiac involvement has been reported as a cause of death in 10–20% of patients with polymyositis [11, 12]. A number of cases of cardiac and skeletal myositis have been reported in the literature in which an underlying cause was identified. Kerr and Spiera reported six cases of myocarditis as a complication in scleroderma patients with myositis. In contrast to our patient, all of the six patients had an elevated CK, and five of them had reduced LVEF [13]. Infectious agents such as influenza virus and toxoplasma have been reported to cause polymyositis and myocarditis in a 4-year-old and a 13-year-old immunocompetent patient, respectively [14, 15]. Greaves et al. investigated the prevalence of myocarditis and skeletal myositis using CK, CK-MB, and troponin I/T in 152 adults with acute viral infection. The study showed that skeletal muscle injury was far more common than cardiac muscle injury in that cohort of patients [16]. Further literature review revealed a recently reported case of myocarditis and skeletal myositis in a 65-year-old following a seasonal influenza vaccination. Similar to our case, his myocarditis was diagnosed with cardiac MRI and an elevated troponin I, but in contrast to our case, his CK level was markedly elevated [17].

Our case is unique as there was no evidence from the history, clinical examination, or extensive laboratory investigations to indicate a specific aetiology of this man's myositis. Endomyocardial biopsy is the gold standard test for myocarditis; however, the clinical presentation in conjunction with the raised troponin level and a cardiac MRI demonstrating myocardial muscle oedema in keeping with myocarditis was considered sufficient to make a diagnosis without a myocardial biopsy, since such a procedure would be invasive and not without significant side effects. Consequently, the patient was noted to have calf muscle symptoms and MRI demonstrated skeletal muscle oedema in a distribution in keeping with skeletal muscle myositis. At this juncture, a skeletal muscle biopsy was proposed but the patient was not happy to consent to this procedure. However, in light of the history and clinical and MRI findings, it was considered reasonable to make a diagnosis of skeletal muscle myositis on a background of myocarditis and to treat the patient accordingly.

The first-line treatment of idiopathic inflammatory myositis is a high dose of corticosteroids (60–100 mg), which should be tapered after 4–6 weeks according to response [18]. Most patients require an additional immunosuppressive agent such as methotrexate, azathioprine, or mycophenolate mofetil [18]. There are very few studies comparing immunosuppressant regimes (azathioprine with methotrexate, cyclosporine with methotrexate, and intramuscular methotrexate with oral methotrexate plus azathioprine). They showed no statistically significant difference in efficacy between the treatment regimes [19]. We chose methotrexate due to our extensive experience with the drug and the fact that no trials have shown the superiority of one agent over the other. Other treatment options such as intravenous immunoglobulins, cyclophosphamide, and rituximab can be used but are reserved for severe or refractory cases [18].

In conclusion, we report a rare case of clinically significant cardiac and skeletal myositis whose cause is unclear. In patients with acute myocarditis, it is important to consider the possibility of an associated skeletal myositis when suggestive symptoms are present. MRI can be useful to establish the diagnosis particularly if laboratory tests are not conclusive. Further trials are needed to determine whether cardiac involvement is associated with certain types and subtypes of myositis or not.
